# Differences in meal patterns and timing with regard to central obesity in the ANIBES (‘Anthropometric data, macronutrients and micronutrients intake, practice of physical activity, socioeconomic data and lifestyles in Spain’) Study

**DOI:** 10.1017/S1368980017000635

**Published:** 2017-04-17

**Authors:** Aránzazu Aparicio, Elena E Rodríguez-Rodríguez, Javier Aranceta-Bartrina, Ángel Gil, Marcela González-Gross, Lluis Serra-Majem, Gregorio Varela-Moreiras, Rosa Maria Ortega

**Affiliations:** 1 VALORNUT Research Group, Department of Nutrition, Faculty of Pharmacy, Complutense University of Madrid, Plaza Ramón y Cajal s/n, 28040 Madrid, Spain; 2 CIBER OBN, Biomedical Research Networking Center for Physiopathology of Obesity and Nutrition, Carlos III Health Institute, Madrid, Spain; 3 Department of Preventive Medicine and Public Health, University of Navarra, Pamplona, Spain; 4 Department of Biochemistry and Molecular Biology II, Institute of Nutrition and Food Sciences, University of Granada, Granada, Spain; 5 ImFINE Research Group, Department of Health and Human Performance, Technical University of Madrid, Madrid, Spain; 6 Research Institute of Biomedical and Health Sciences & Medical School, University of Las Palmas de Gran Canaria, Edificio Departamental y de Investigación, Las Palmas de Gran Canaria, Las Palmas, Spain; 7 Department of Pharmaceutical and Health Sciences, Faculty of Pharmacy, CEU San Pablo University, Madrid, Spain; 8 Spanish Nutrition Foundation (FEN), Madrid, Spain

**Keywords:** Waist-to-height ratio, Obesity, Central obesity, Timing, Variety

## Abstract

**Objective:**

To study the association of meal patterns and timing with central obesity to identify the best dietary strategies to deal with the increasing obesity prevalence.

**Design:**

A cross-sectional study performed on data from a representative sample of the Spanish population. Height and waist circumference were measured using standardized procedures and waist-to-height ratio (WHtR) was calculated. The sample was divided into those without central obesity (WHtR<0·5) and those with central obesity (WHtR≥0·5).

**Setting:**

ANIBES (‘Anthropometric data, macronutrients and micronutrients intake, practice of physical activity, socioeconomic data and lifestyles in Spain’) Study.

**Subjects:**

Adults aged 18–64 years (*n* 1655; 798 men and 857 women).

**Results:**

A higher percentage of people ate more than four meals daily in the group without central obesity and those with central obesity more frequently skipped the mid-afternoon snack than those without. Breakfasts containing >25 % of total energy intake and lunches containing >35 % of total energy intake were associated with increased likelihood of central obesity (OR=1·874, 95 % CI 1·019, 3·448; *P*<0·05 and OR=1·693, 95 % CI 1·264, 2·268; *P*<0·001, respectively). On the contrary, mid-morning snacks and mid-afternoon snacks containing >15 % of total energy were associated with decreased likelihood of central obesity (OR=0·477, 95 % CI 0·313, 0·727; *P*<0·001 and OR=0·650, 95 % CI 0·453, 0·932; *P*<0·05, respectively). The variety of cereals, wholegrain cereals and dairy was higher in the population without central obesity.

**Conclusions:**

Our results suggest that ‘what and when we eat’ should be considered dietary strategies to reduce central obesity.

The prevalence of obesity is increasing worldwide^(^ ^1^ ^)^ and the role of individual dietary components has been the focus of considerable research in the field of obesity^(^ ^2^ ^–^ ^4^ ^)^.

Changes in diet and physical activity are essential treatments in the strategies to reduce excess weight^(^ ^5^ ^)^; however, not all of these are equally effective^(^ ^6^ ^)^. It has been proposed that some types of diets (low-calorie diets, diets with different proportions of fat, protein and carbohydrates, traditional healthy eating patterns, etc.) may improve risk factors associated with obesity; however, each diet has limitations, ranging from high dropout rates to maintenance difficulties. In addition, most of these dietary regimens have the ability to attenuate some, but not all, of the components involved in this complicated multifactorial condition. In its 2013 guidelines, the Canadian Diabetes Association reviewed the efficacy of some of the more prominent dietary patterns or diets. The conclusion was that dietary patterns including vegetarian, the Mediterranean and the Dietary Approaches to Stop Hypertension diets could be recommended. In addition, certain popular weight-loss diets (Atkins, Protein Power Plan, Ornish, Weight Watchers and Zone) had sufficient evidence to suggest their use by people with diabetes whose lifestyles and personal preferences were congruent with the diets^(^ ^7^ ^)^. Having in mind this situation, it is currently unknown which intervention is the more correct and interest has arisen in the time of day foods are consumed (food timing)^(^ ^8^ ^)^.

Diverse studies show inconsistent findings between BMI and dietary patterns. It seems that the adoption of a dietary pattern characterized by high intakes of red and processed meats, refined grains, sweets and desserts (Western pattern) is associated with larger weight gain, whereas a dietary pattern usually characterized by high intakes of fruits, vegetables, whole grains, fish and poultry (healthy pattern) may facilitate weight maintenance^(^ ^9^ ^)^ and have fewer metabolic consequences^(^ ^10^ ^,^ ^11^ ^)^.

On the other hand, recent studies conducted in man suggest that eating at the right or wrong time, restricting eating hours, time allocation for meals, timing of macronutrient consumption during the day and even variety of the diet may also have an important role in total energy intake and therefore in the regulation of adiposity and body weight^(^ ^12^ ^–^ ^14^ ^)^. It has been observed that characteristics of dietary behaviour such as skipping breakfast^(^ ^15^ ^)^, eating more of the day’s total energy intake during the evening^(^ ^16^ ^)^, higher frequency of meals eaten away from home^(^ ^17^ ^)^ and higher eating and snack frequency^(^ ^18^ ^)^ are associated with a higher risk of being overweight/obese or having adverse metabolic consequences^(^ ^10^ ^,^ ^11^ ^,^ ^19^ ^)^. Nevertheless, because it is a new aspect to explore and few studies have been performed, especially in relation to the condition of central obesity, further research is needed.

In view of the above, the aim of our research was to study the association of eating frequency, timing of nutrient intake and meal patterns with central obesity in order to identify the best dietary strategies to deal with the increasing prevalence of obesity.

## Experimental methods

The design, protocols and methodologies of the ANIBES (‘Anthropometric data, macronutrients and micronutrients intake, practice of physical activity, socioeconomic data and lifestyles in Spain’) Study have been described in detail elsewhere^(^ ^20^ ^–^ ^22^ ^)^. Briefly, the study was performed to record food and beverage intakes, dietary habits and anthropometric data, as well as energy expenditure and physical activity patterns of the Spanish population.

### Participants

The ANIBES Study was conducted on a representative Spanish population. The sample for the ANIBES Study was designed based on 2012 census data published by the INE (Instituto Nacional de Estadística/Spanish Bureau of Statistics). The total sample size was calculated based on a 0·05 probability of Type I error (rejecting a null hypothesis when it is true) and 0·1 probability of Type II error (accepting a null hypothesis when it is wrong) in the main outcome of the study (energy intake). Sampling was performed in 128 random regions all over Spain.

For the sampling, the following variables were taken into account: age group (children (9–12 years), adolescents (13–17 years), adults (18–64 years) and seniors (65–75 years)); adult group (young adults (18–30 years), middle adults (31–49 years) and old adults (50–64 years)); sex; geographical distribution (Northeast, Levant, South, West, North-Central, Barcelona, Madrid, and Balearic and Canary Islands); and locality size (2000–30 000 inhabitants, rural population; 30 000–200 000 inhabitants, semi-urban population; and >200 000 inhabitants, urban population). Geographical distributions were grouped into four different regions (Centre, Atlantic, Mediterranean and South)^(^ ^20^ ^)^.

The final study sample comprised 2009 individuals aged 9–75 years (1013 males, 50·4 %; 996 females, 49·6 %). The present investigation is focused on the adult population (excluded elderly) aged 18–64 years (*n* 1655; 798 men, 857 women). Data were collected between mid-September and mid-November 2013. Participants were asked to sign the letter of consent for participation in the study as has been described in detail elsewhere^(^ ^20^ ^)^.

Several exclusion criteria were applied: individuals living in an institutional setting (e.g. colleges, nursing homes, hospitals and others); individuals following a therapeutic diet owing to recent surgery or taking any medical prescription; potential participants with a transitory illness (i.e. flu, gastroenteritis, chicken pox) at the time of the fieldwork; and individuals employed in areas related to consumer science, marketing or the media^(^ ^20^ ^–^ ^22^ ^)^.

### Methods

#### Diet

Dietary data collection methods have been described elsewhere^(^ ^20^ ^–^ ^22^ ^)^. Dietary intake was assessed via face-to-face 24 h recall (1d intake, not included in the final data) and a 3d record using a tablet device (Samsung Galaxy Tab 2 7.0) on two weekdays and one weekend day, including information on all foods and beverages consumed at home and away, as well as eating habits (e.g. recipes, brands, types of milk and fat spread usually consumed, among other data). The participants had to take pictures of their meal before and after they ate so we could know the exactly amount of food they consumed. The number of meals eaten away from home was calculated using the 3d record questionnaire.

A manual of procedures to facilitate food collection was provided to participants, in addition to a toll-free telephone number in case they had any questions regarding the software, use of the device or the food and beverage record. Food, beverage, energy and nutrient intakes were calculated using software (VD-FEN 2.1) that was newly developed for the ANIBES Study by the Spanish Nutrition Foundation and is based mainly on expanded and updated Spanish food composition tables^(^ ^23^ ^)^. Food and beverage consumption data (g/d) were categorized into thirty-eight food groups. A modified version of the measure developed by Murphy *et al*.^(^ ^24^ ^)^ was used to assess dietary variety. In our study, to determine the number of servings of foods consumed by participants, the number of grams of each food or beverage consumed was divided by the Spanish standard servings^(^ ^25^ ^)^. Although initially foods and beverages were classified into thirty-eight groups, these were regrouped according to the Spanish Guideline ‘La Pirámide de Alimentación Saludable’ (Healthy Eating Pyramid)^(^ ^25^ ^)^. Dietary variety was defined as the different number of foods in each food group consumed. A score of 1 point was assigned for intake of at least one-half serving of each food group over the 3d period. Total final dietary variety was calculated by adding the scores obtained in each group of foods.

The 3d food record included some columns to indicate the exact time that it was used in each meal of the day (e.g. start breakfast: 08.30 hours, finish breakfast: 08.42 hours). Also, data collection was structured according to the occasions of food intake: breakfast, mid-morning meal, lunch, mid-afternoon meal and dinner. Energy consumed in each meal was compared with the theoretical energy that each of them should provide according to what is considered a healthy diet^(^ ^26^ ^)^.

#### Physical activity level

Physical activity was estimated based on the International Physical Activity Questionnaire^(^ ^27^ ^)^. Time spent in vigorous-intensity physical activity was calculated and grouped as <75 min/week, 75–150 min/week, 151–300 min/week or >300 min/week. Also, moderate- to vigorous-intensity physical activity was calculated and grouped as <150 min/week, 151–300 min/week or >300 min/week, based on public health guidelines^(^ ^28^ ^)^. Information about the time spent sleeping was obtained from this questionnaire.

#### Body measurements

Height and waist circumference were measured using standardized procedures by well-trained interviewers to minimize the inter-observer CV^(^ ^29^ ^)^. Height was measured in triplicate using a Seca^®^ model 206 stadiometer (Medizinische Messsysteme und Waagen seit 1840, Hamburg, Germany; range 70–205 cm, precision 1 mm). Waist circumference was assessed in triplicate using a Seca^®^ 201 tape measure (Seca, Hamburg, Germany; range 0–150 cm, precision 1 mm). General adiposity was assessed using central obesity as obtained from waist-to-height ratio (WHtR): WHtR=waist circumference (cm)/height (cm). According to WHtR, participants were classified into two categories; specifically, those without central obesity (WHtR<0·5) and those with central obesity (WHtR≥0·5)^(^ ^30^ ^–^ ^33^ ^)^.

### Statistical analysis

Data are presented as means, standard deviations and percentages. Analyses were performed using the statistical software package IBM SPSS Statistics Version 22.0. The Kolmogorov–Smirnoff test was used to determine whether the variables followed a normal distribution to decide between parametric or non-parametric analysis. Differences between groups were performed using the Student *t* test or Mann–Whitney *U* test. The *z* test was used to compare proportions. The effects of sex, age and other covariables such as energy intake on risk of central adiposity were analysed via logistic regression analysis to calculate the odds ratios. The dependent variable was WHtR. Reference groups comprised individuals without central adiposity (WHtR<0·5). The 95 % confidence intervals were calculated and Wald’s test used for comparison of the odds ratios. Significance was set at *P*<0·05.

## Results

Dietary characteristics of the Spanish population according to sex are shown in Tables 1 and 2. Women ate more meals per day than men; specifically, 54·4 % of women ate more than four meals daily (Table 1). The percentage of men who skipped breakfast, mid-morning snack and mid-afternoon snack was higher than in women. Men consumed more energy after 14.00 hours and more energy from dinner than women, and women spent more time eating all meals, mid-afternoon snack and breakfast than men (Table 1). Finally, the variety of meat and eggs was higher in men than women, but the variety of fish, fruit, wholegrain cereals and dairy was higher in women (Table 2).

**Table 1:** Diet characteristics of the studied population according to sex; representative sample of Spanish adults aged 18–64 years, ANIBES (‘Anthropometric data, macronutrients and micronutrients intake, practice of physical activity, socioeconomic data and lifestyles in Spain’) Study

	Total (*n* 1655)		Men (*n* 798)		Women (*n* 857)	
	Mean or %	sd	Mean or %	sd	Mean or %	sd
No. of eating occasions per 24 h	4·11	0·85	3·95***	0·84	4·26***	0·83
No. of meals daily (%)						
0–3	15·6	–	20·4*	–	11·1*	–
3–4	37·5	–	40·7*	–	34·5*	–
>4	46·9	–	38·8*	–	54·4*	–
No. of meals away from home	1·08	0·92	1·05	0·93	1·11	0·92
Percentage of individuals who skip meals (%)						
Breakfast	1·99	–	3·01*	–	1·05*	–
Mid-morning snack	34·90	–	38·70*	–	31·40*	–
Lunch	0·60	–	0·00	–	0·12	–
Mid-afternoon snack	29·80	–	36·10*	–	23·90*	–
Dinner	0·30	–	0·25	–	0·35	–
Time spent on each meal of the day (min/d)						
Breakfast	12·5	8·9	11·9***	9·4	13·0***	8·3
Mid-morning snack	4·9	7·8	4·8	7·6	5·1	8·0
Lunch	19·6	10·4	19·3	10·0	19·9	10·7
Mid-afternoon snack	6·1	9·9	5·1***	9·3	7·0***	10·4
Dinner	18·7	10·7	18·7	10·9	18·8	10·5
Total time	64·1	29·8	62·3**	29·8	65·8**	29·6
Total energy (kJ/d)	7598	2142	8226***	2272	7008***	1828
Total energy (kcal/d)	1816	512	1966***	543	1675***	437
Percentage of energy consumed at each meal (%)						
Breakfast	16·4	8·5	15·5***	8·7	17·2***	8·3
Mid-morning snack	4·6	6·2	4·8	6·8	4·4	5·5
Lunch	39·8	9·9	39·8	5·7	39·5	9·9
Mid-afternoon snack	5·5	6·4	4·8***	6·4	6·1***	6·4
Dinner	30·5	9·9	31·7***	9·9	29·4***	9·8
Evening:morning energy intake ratio (cut point at 14.00 hours)	4·50	19·70	4·65*	10·31	4·35*	25·49
Time spent sleeping (h)	7·46	1·13	7·46	1·11	7·46	1·12

*P<0·05, **P<0·01, ***P<0·001 (significantly different between men and women). The Student *t* test (or the Mann–Whitney *U* test if the distribution of results was not homogeneous) was used to compare variables between men and women. The *z* test was used to compare proportions.

**Table 2:** Diet variety of the studied population according to sex; representative sample of Spanish adults aged 18–64 years, ANIBES (‘Anthropometric data, macronutrients and micronutrients intake, practice of physical activity, socioeconomic data and lifestyles in Spain’) Study

	Total		Men		Women	
	Mean	sd	Mean	sd	Mean	sd
Total variety	26·9	6·7	27·0	7·0	26·8	6·4
Meat variety	2·96	1·75	3·18***	1·86	2·76***	1·62
Fish variety	1·28	1·42	1·22**	1·44	1·34**	1·39
Egg variety	0·65	0·51	0·68*	0·51	0·63*	0·51
Vegetables variety	5·02	2·47	4·88	2·48	5·14	2·46
Fruit variety	1·73	1·75	1·58***	1·68	1·88***	1·81
Vegetables and fruit variety	6·75	3·40	6·46	3·39	7·02	3·39
Cereals variety	4·05	1·84	3·97	1·76	4·13	1·91
Whole cereals variety	0·26	0·55	0·20***	0·48	0·32***	0·60
Legumes variety	0·50	0·67	0·53	0·69	0·48	0·64
Dairy variety	2·78	1·61	2·70*	1·60	2·85*	1·62

*P<0·05, **P<0·01, ***P<0·001 (significantly different between men and women). The Student *t* test (or the Mann–Whitney *U* test if the distribution of results was not homogeneous) was used to compare variables between men and women.

Dividing the population according to the presence of central obesity (WHtR≥0·5), there were no differences in the total number of meals eaten per day but the percentage of people eating more than four meals daily was higher in the group without central obesity (Table 3). Furthermore, eating four or more meals daily was associated with reduced likelihood of central obesity in men, after adjusting for age and energy intake (OR=0·684, 95 % CI 0·479, 0·977; *P*=0·037). Those with central obesity more frequently skipped the mid-afternoon snack, spent less time on the mid-morning snack and spent more time on lunch than those without central obesity (true for the general population and men). Men with central obesity spent more time eating all meals as a sum than those without central obesity (Table 3).

**Table 3:** Diet characteristics of the studied population according to central obesity classification†; representative sample of Spanish adults aged 18–64 years, ANIBES (‘Anthropometric data, macronutrients and micronutrients intake, practice of physical activity, socioeconomic data and lifestyles in Spain’) Study

	Total				Men				Women			
	WHtR<0·5 (*n* 689)		WHtR≥0·5 (*n* 966)		WHtR<0·5 (*n* 282)		WHtR≥0·5 (*n* 516)		WHtR<0·5 (*n* 407)		WHtR≥0·5 (*n* 450)	
	Mean or %	sd	Mean or %	sd	Mean or %	sd	Mean or %	sd	Mean or %	sd	Mean or %	sd
No. of eating occasions per 24 h	4·14	0·85	4·09	0·85	3·98	0·86	3·94	0·83	4·24	0·83	4·27	0·84
No. of meals daily (%)												
0–3	15·5	–	15·6	–	21·3	–	20·0	–	11·5	–	10·7	–
3–4	34·5*	–	39·6*	–	35·8	–	43·4	–	33·7	–	35·3	–
>4	49·9*	–	44·7*	–	42·9	–	36·6	–	54·8	–	54·0	–
No. of meals away from home	1·21***	0·93	0·99***	0·90	1·12	0·93	1·02	0·92	1·28***	0·94	0·96***	0·87
Percentage of individuals who skip meals (%)												
Breakfast	2·61	–	1·55	–	3·55	–	2·71	–	1·97	–	0·22	–
Mid-morning snack	32·80	–	36·44	–	34·75	–	40·89	–	31·45	–	31·33	–
Lunch	0·15	–	0·00	–	0·00	–	0·00	–	0·25	–	0·00	–
Mid-afternoon snack	26·56*	–	32·09*	–	32·98	–	37·79	–	22·11	–	25·56	–
Dinner	0·44	–	0·21	–	0·35	–	0·19	–	0·49	–	0·22	–
Time spent on each meal of the day (min/d)												
Breakfast	12·1	8·7	12·7	9·0	11·7	9·8	12·0	9·2	12·4	8·0	13·5	8·6
Mid-morning snack	5·1*	7·6	4·8*	7·9	4·9	7·0	4·7	7·9	5·3	8·1	4·9	8·0
Lunch	19·1**	10·6	20·0**	10·2	18·9	10·9	19·5	9·6	19·2*	10·5	20·5*	10·9
Mid-afternoon snack	6·0	8·9	6·2	10·5	5·4	9·0	5·0	9·5	6·4	8·9	7·6	11·5
Dinner	19·1	11·1	18·5	10·4	18·7	10·7	18·7	11·0	19·3	11·4	18·3	9·7
Total time	63·7	29·7	64·4	29·8	60·8**	30·7	63·1**	29·2	65·7	28·8	66·0	30·4
Total energy (kJ/d)	7891***	2272	7385***	2017	8795***	2431	7916***	2121	7263***	1924	6778***	1703
Total energy (kcal/d)	1886***	543	1765***	482	2102***	581	1892***	507	1736***	460	1620***	407
Percentage of energy consumed at each meal (%)												
Breakfast	16·1	8·3	16·6	8·7	15·6	8·3	15·4	8·8	16·5*	8·21	17·9*	8·4
Mid-morning snack	5·2***	6·6	4·1***	5·9	5·8**	7·3	4·2**	6·5	4·8	6·0	4·0	5·1
Lunch	38·0***	9·6	41·1***	9·8	37·6***	9·3	41·6***	9·8	38·3***	9·9	40·5***	9·7
Mid-afternoon snack	6·3***	6·9	4·9***	6·1	6·0***	7·2	4·2***	5·8	6·5*	6·6	5·8*	6·2
Dinner	30·6	10·2	30·4	9·7	31·8	10·0	31·7	9·9	29·8	10·3	29·0	9·2
Evening:morning energy intake ratio (cut point at 14.00 hours)	3·50	3·00	5·21	25·66	3·56	2·97	5·25	12·61	3·45*	3·02	5·16*	35·06
Time spent sleeping (h)	7·62***	1·08	7·34***	1·15	7·64***	0·99	7·35***	1·14	7·60***	1·14	7·33***	1·17

WHtR, waist-to-height ratio. *P<0·05, **P<0·01, ***P<0·001 (significantly different between WHtR<0·5 and WHtR≥0·5). The Student *t* test (or the Mann–Whitney *U* test if the dis*t*ribution of results was not homogeneous) was used to compare variables between WHtR<0·5 and WHtR≥0·5. The *z* test was used to compare proportions.

† Without central obesity, WHtR<0·5; with central obesity, WHtR≥0·5.

People with central obesity consumed fewer meals away from home, slept for shorter, and ate more energy at lunch and less energy at the mid-morning and mid-afternoon snacks than those without this type of obesity (Table 3). Specifically, lunches containing more than 35 % of total daily energy were associated with increased likelihood of central obesity, adjusting for sex and age (OR=1·693, 95 % CI 1·264, 2·268; *P*<0·001). On the contrary, mid-morning snacks and mid-afternoon snacks containing more than 15 % of total daily energy were associated with decreased likelihood of central obesity (OR=0·477, 95 % CI 0·313, 0·727; *P*<0·001 and OR=0·650, 95 % CI 0·453, 0·932; *P*<0·05, respectively). This situation was also observed in men (Table 3), where mid-morning snacks contributing 10–15 % and more than 15 % to total daily energy intake, and mid-afternoon snacks contributing more than 15 % to total daily energy intake, were associated with decreased likelihood of central obesity (OR=0·492, 95 % CI 0·295, 0·819; *P*<0·01; OR=0·546, 95 % CI 0·333, 0·893; *P*<0·05; OR=0·430, 95 % CI 0·260, 0·709; *P*<0·001, respectively). In women, those with central obesity ate more energy at breakfast and lunch and less energy at mid-afternoon snack than those without central obesity. Specifically, breakfasts containing more than 25 % of total daily energy were associated with increased likelihood of central obesity (OR=1·874, 95 % CI 1·019, 3·448; *P*<0·05). Nevertheless, women with central obesity ate more energy after 14.00 hours than women without central obesity (Table 3).

Dietary variety was higher in the total population and men without central obesity. Specifically, the variety of cereals, wholegrain cereals and dairy was higher in the population without central obesity and of dairy in women without central obesity (Table 4).

**Table 4:** Diet variety of the studied population according to central obesity classification†; representative sample of Spanish adults aged 18–64 years, ANIBES (‘Anthropometric data, macronutrients and micronutrients intake, practice of physical activity, socioeconomic data and lifestyles in Spain’) Study

	Total				Men				Women			
	WHtR<0·5		WHtR≥0·5		WHtR<0·5		WHtR≥0·5		WHtR<0·5		WHtR≥0·5	
	Mean	sd	Mean	sd	Mean	sd	Mean	sd	Mean	sd	Mean	sd
Total variety	27·6***	6·8	26·4***	6·5	28·0***	7·1	26·3***	6·8	27·2	6·5	26·4	6·1
Meat variety	2·97	1·71	2·95	1·78	3·24	1·84	3·14	1·87	2·78	1·59	2·74	1·65
Fish variety	1·28	1·45	1·28	1·39	1·20	1·44	1·23	1·45	1·34	1·45	1·34	1·32
Egg variety	0·64	0·51	0·66	0·51	0·70	0·51	0·68	0·50	0·60	0·51	0·65	0·51
Vegetables variety	5·12	2·55	4·94	2·41	5·08	2·61	4·77	2·40	5·14	2·52	5·14	2·41
Fruit variety	1·80	1·85	1·68	1·68	1·72	1·87	1·50	1·56	1·86	1·83	1·89	1·80
Vegetables and fruit variety	6·92	3·58	6·63	3·26	6·79	3·74	6·28	3·17	7·01	3·47	7·03	3·31
Cereals variety	4·18*	1·89	3·96*	1·81	4·11	1·71	3·89	1·79	4·23	2·00	4·04	1·83
Whole cereals variety	0·31*	0·61	0·23*	0·50	0·23	0·52	0·19	0·47	0·37	0·66	0·28	0·53
Legumes variety	0·51	0·68	0·50	0·65	0·55	0·72	0·52	0·68	0·49	0·65	0·47	0·63
Dairy variety	2·97***	1·62	2·65***	1·60	2·80	1·59	2·65	1·60	3·08***	1·63	2·65***	1·59

WHtR, waist-to-height ratio. *P<0·05, ***P<0·001 (significantly different between WHtR<0·5 and WHtR≥0·5). The Student *t* test (or the Mann–Whitney *U* test if the distribution of results was not homogeneous) was used to compare variables between WHtR<0·5 and WHtR≥0·5.

† Without central obesity, WHtR<0·5; with central obesity, WHtR≥0·5.

## Discussion

Some studies have shown sex differences in eating patterns^(^ ^34^ ^,^ ^35^ ^)^ and that, in general, women are more likely to find healthy eating more important^(^ ^19^ ^,^ ^36^ ^)^. Studies conducted in Ireland reported that women were generally more prone to make conscious efforts to try to eat a healthy diet ‘most of the time’, while men were three times more likely to ‘hardly ever’ make such conscious efforts to eat a healthy diet^(^ ^37^ ^,^ ^38^ ^)^. These results are consistent with findings in our work in which women followed more adequate dietary habits than men, eating a greater number of meals daily, skipping fewer meals, taking more time on those meals and eating more energy in the morning than in the evening. In addition, our study also found that women had a greater variety of foods considered healthy in their diets than men, such as fish, fruits, whole grains and dairy. This situation has also been described by other authors who found that women often report to have a lower intake of fat and higher intakes of fruits and vegetables and dietary fibre^(^ ^19^ ^,^ ^36^ ^)^, and that women are more likely than men to choose or avoid foods following concerns about health and, accordingly, choose or avoid foods due to their contents^(^ ^39^ ^)^.

Although other authors have reported that meal and snack patterns, including the daily eating frequency, affect body condition and the development of chronic diseases^(^ ^19^ ^,^ ^34^ ^,^ ^40^ ^–^ ^42^ ^)^, others have not found consistent results^(^ ^43^ ^,^ ^44^ ^)^. Thus this is a controversial topic and must be investigated.

In the present study, when comparing people and specifically women according to central obesity (measured by WHtR), the number of meals eaten away from home was greater in those without central obesity. This result contrasts with other studies, which found overweight and obesity to be associated with the frequency of eating away from home^(^ ^45^ ^,^ ^46^ ^)^. Nevertheless, a study carried among 1070 housewives from Korea found that choosing healthy meals away from home was more important for housewives than refraining from eating out^(^ ^47^ ^)^. Similarly, a recent study using Brazilian data indicated that eating out was associated with overweight and obesity only among men, whereas among women, eating sit-down meals outside the home did not cause obesity, suggesting that women make healthier food choices when they eat outside the home^(^ ^48^ ^)^.

In the present study, the percentage of people who ate four or more meals daily was greater in the group without central obesity. Furthermore, eating four or more meals daily was associated with reduced likelihood of central obesity in men, after adjusting for age and energy intake. Similarly, in a study including 1355 men and 1654 women between 47 and 68 years of age, eating three or fewer meals daily (compared with eating six or more meals daily) was associated with increased likelihood of central obesity (waist circumference ≥102 cm), when adjusting for total energy intake, lifestyle and dietary factors, in men (OR=2·09, 95 % CI 1·03, 4·27; *P*=0·043)^(^ ^19^ ^)^. In another study carried out in 191 overweight Hispanic youths (8–18 years), those who consumed three or more eating occasions (≥209 kJ (≥50 kcal) and ≥15 min from any prior eating occasion) had 9 % lower BMI *Z*-score (*P*<0·01), 9 % lower waist circumference (*P*<0·01), 29 % lower fasting insulin (*P*=0·02), 31 % lower HOMA-IR (homeostasis model assessment of insulin resistance) values (*P*=0·02) and 19 % lower TAG (*P*<0·01) compared with those who consumed fewer than three eating occasions, although the former consumed 23 % more energy per day than the latter (*P*<0·01)^(^ ^49^ ^)^. The authors stated that one possible reason to explain the relationship between a high frequency of meals and a lower rate of obesity could be due to a lower increase of fasting insulin values, as well as decreased insulin resistance, as has also been shown in other studies^(^ ^50^ ^–^ ^52^ ^)^, because insulin inhibits lipase enzyme activity and increases fat deposition. Thus, the present study suggests that there may be a favourable impact of increasing eating frequency with regard to preventing central obesity.

The importance of not skipping any of the four or five meals that are recommended daily has been widely studied. In one of the first studies performed, in which thirty-five individuals were instructed to keep a continuous record of their eating behaviour during a 10-week behavioural weight-loss programme, Schlundt *et al*.^(^ ^53^ ^)^ found an association between meal skipping and overeating at subsequent meals. In this respect, several investigations have found that skipping breakfast is associated with increased prevalence of general and central obesity^(^ ^15^ ^,^ ^42^ ^,^ ^54^ ^,^ ^55^ ^)^, highlighting the importance of this meal. Nevertheless, although we did not find this association in the present study, we observed that consuming breakfast with an energy content greater than 25 % of daily energy intake was associated with increased likelihood of central obesity, so it is important take care with the energy content of breakfast.

Similarly, skipping the mid-afternoon snack was associated with increased likelihood of central obesity in the present study. Furthermore, the energy content of this meal must be similar to that recommended (15 % of total daily energy intake). The importance of the mid-morning and mid-afternoon snacks on obesity has been less studied in the literature than breakfast and the results found have been contradictory, probably owing to the different dietary patterns between countries^(^ ^56^ ^)^. In accordance with our results, when studying 1314 participants aged 20–79 years from four Spanish cities, Keller *et al*.^(^ ^57^ ^)^ found that having an afternoon meal was negatively associated with central obesity (waist circumference: ≥88 cm in women and ≥102 cm in men), after adjusting for all confounders (OR=0·60; 95 % CI 0·41, 0·88; *P*<0·05). Nevertheless, Kong *et al*.^(^ ^58^ ^)^ studied 123 overweight-to-obese postmenopausal women enrolled in two dietary weight-loss programmes during 12 months and found that women who reported mid-morning snacking lost significantly less weight (OR=7·0; 95 % CI 4·1, 9·8) compared with those who were not mid-morning snackers (OR=11·5; 95 % CI 10·2, 12·7; *P*<0·005). This could be because, more than a mid-morning snack, it was an additional ‘pecking’, near the mealtime, that could contribute to excess energy intake.

Having in mind our results, the importance of mid-morning and mid-afternoon snacks having adequate energy content is remarkable because: (i) the foods consumed during these meals could promote a healthier diet, because of their relationship with the consumption of milk and dairy products, fruits and vegetables^(^ ^58^ ^,^ ^59^ ^)^ and because it seems that a balanced mid-morning and mid-afternoon snack may contribute significantly to an adequate daily intake of nutrients^(^ ^60^ ^)^; and (ii) these meals might affect the subsequent eating occasion, and thus lead to lower consumption of foods and energy in lunch and dinner, this situation being beneficial against the risk of obesity and other adverse metabolic consequences^(^ ^10^ ^,^ ^11^ ^,^ ^61^ ^,^ ^62^ ^)^. In fact, according to these results, in the present study people with central obesity had lunch with more energy than people without this condition probably because the mid-morning snack was more inadequate, as explained before. This highlights the importance of not skipping mid-morning and mid-afternoon snacks containing an adequate energy percentage (15 % of the total daily intake) and foods with elevated nutritional quality, as fruits, dairy and fibre-rich foods^(^ ^58^ ^)^. This is endorsed with our results, where a greater variety of foods from cereals, whole grains and dairy was observed in the diet of individuals without central obesity.

Moreover, it has also been observed that the consumption of larger food amounts in the afternoon and evening increases the risk of developing obesity^(^ ^42^ ^,^ ^63^ ^,^ ^64^ ^)^ and impairs weight loss in overweight/obesity^(^ ^65^ ^)^. In the present study, women with central obesity consumed more energy after 14.00 hours (compared with before 14.00 hours) than women without central obesity. In relation to the timing of lunch, in a sample of 420 individuals who followed a 20-week weight-loss treatment, late eaters (lunch time after 15.00 hours) lost less weight than early eaters (lunch time before 15.00 hours). Furthermore, the former had a significantly lower percentage of their total daily energy intake during breakfast and skipped breakfast more frequently than early eaters, effects that could be contributing to the differences in weight loss with lunch timing^(^ ^65^ ^)^. Thus, it is desirable to consume an early lunch in order to prevent the occurrence of obesity and central obesity owing to the possible influence on levels of circulating satiety hormones, such as leptin or ghrelin, by circadian misalignment, that could influence energy intake and expenditure^(^ ^42^ ^,^ ^66^ ^)^.

Several studies show that the speed of eating has a positive association with obesity because eating fast may lead to greater energy intake before the internal signals of satiation, which would have an effect on the weight of a person^(^ ^67^ ^,^ ^68^ ^)^. In the present study, people with central obesity spent less time eating the mid-morning snack than those without central obesity. Nevertheless, some studies found no relationship between eating rate and energy intake^(^ ^69^ ^–^ ^71^ ^)^ and one study observed a higher energy intake with more pauses within meals^(^ ^72^ ^)^. Similarly, in our study, men with central obesity spent more time eating all meals, and women spent more time eating lunch, than those without central obesity, which could be because this longer time allows a greater amount of food to be consumed compared with those who spend less time eating.

Finally, in the present study, people with central obesity slept for less time than those without central obesity. These data agree with other studies that found an association between shorter sleeping time and the risk of obesity^(^ ^73^ ^)^ and abdominal obesity^(^ ^74^ ^)^. Some of the proposed mechanisms to explain the relationship between sleep and obesity suggest that lower leptin and elevated ghrelin levels associated with shorter sleep^(^ ^75^ ^)^ can stimulate appetite and cause weight gain^(^ ^76^ ^)^.

Although ANIBES data were representative of the Spanish population, caution should be attended because the cross-sectional design makes it impossible to determine reverse causality (i.e. obese individuals eat fewer meals daily as a strategy for weight loss).

## Conclusions

In conclusion, the present results suggest that dietary strategies to reduce central obesity could be: consume at least four meals daily, with a breakfast containing less than 25 % of total daily energy intake; include a mid-morning and a mid-afternoon snack in the diet (which provide at least 15 % of total daily energy intake); have lunch at an appropriate time (about 14.00 hours) and with an energy contribution not exceeding 35 % of total daily energy intake; and include the maximum number of foods belonging to the groups of dairy products, cereals and whole grains.
